# Perverted: The History of Homœopathy

**Published:** 1881-05

**Authors:** 


### HOMŒOPATHIC PHYSICIAN.

A MONTHLY JOURNAL OF MEDICAL SCIENCE.

"If our school ever gives up the strict inductive method of Hahnemann, we are lost, and deserve to be mentioned only as a caricature, in the history of medicine." — CONSTANTINE HERING.

Vol. I. MAY, 1881. No. 5.

### EDITORIAL.

PERVERTED: THE HISTORY OF HOMŒOPATHY.—In the "Historical volume of the World's Homœopathic Convention held in Philadelphia, 1876, under the auspices of the American Institute of Homœopathy," there are many errors. It was to be expected, from the confused condition of homœopathic statistics, that there would be many mistakes, omissions, etc., in such a work. But, in the history of homœopathy in the City of Philadelphia, these errors are too glaring and too one-sided to be unintentional. *Unintentional or accidental errors do not all tend the one way, nor alter only those facts which the historian may have an interest in perverting!*

The faculties of the Homœopathic College of Pennsylvania have been moulded to suit the æsthetic taste of the historian. But the greatest perversion—-wilful perversion—which disgraces this volume and insults every homœopathist and every *homœopathic college*, is the attempt to palm off the Penn Medical University as a homœopathic medical school. See "Historical Volume," p. 801.

A short sketch of this "university" will be interesting to the members of the American Institute of Homœopathy, under whose "auspices" the history is published, and under whose seal it goes forth as though stamped with its approbation.

The Penn Medical University was chartered in 1853, not as a homœopathic medical school, but simply as a medical school, and empowered to grant diplomas in medicine after two courses of study. It was but a re-organization of the Pennsylvania University of Medicine, which closed in 1852 for and good satisfactory reasons. After the session of 1863–4, the most gifted members of its learned faculty abandoned the Penn Medical University for the seemingly broader platform of the Eclectic Medical College of Philadelphia. A queer proceeding for *professors* of *homœopathy!* No sooner were these restive spirits safely housed in their new temple of Aesculapius, than they became aware that the term "*eclectic*" in their title was "*restrictive*" and obnoxious, hence they hied themselves to Harrisburg and obtained a new charter (charters are easily obtained there). These honest spirits, desirous of being fair and just, threw in another college with the Eclectic Medical College of Philadelphia, and thus read their new charter: "Be it enacted by the Senate and House of Representatives of the commonwealth of Pennsylvania, in general assembly met, and it is hereby enacted by the authority of the same, that the corporate title of the said, the American College of Medicine, in Pennsylvania and the Eclectic Medical College of Philadelphia, be and the same is hereby changed, and the said corporation shall hereafter be known by the name, style and title of the Philadelphia University of Medicine and Surgery." (Transactions of Pennsylvania Legislature for 1865, p. 469).

The celebrated Dr. Paine was dean of these notorious institutions! These various changes scarcely befit an *honorable* homœopathic medical school.

But again, we read in this history (p. 802), "Its [The Penn Medical University] last course of lectures was given in the winter of 1863–4. The influence of the war, which was seriously felt by all the medical schools of the country, led to the closing of its doors at that time." This institution did not close its doors *for good* in 1863–4; it exists *now, this time* under a *nominal* homœopath, and its reputation is neither homœopathic nor good. The following "notice" in *The Record* (Phila)., proves its present existence:*

Notice is hereby given that application will be made to the Legislature of Pennsylvania at its session of 1881 for the passage of a bill, to be entitled "An Act to annul the charter of the Penn Medical College of Philadelphia, otherwise called the Penn Medical University," the object of which is to annul and repeal the said charter and to dissolve said corporation.

JOHN NORRIS.

January 31, 1881.

To prove the bad reputation this place had and now has, we quote from the same newspaper under date of March 25th, 1881.

As a result of *The Record's* expose of Buchanan's business the charters of the American University of Philadelphia, the Eclectic Medical College of Pennsylvania, the Philadelphia University of Medicine and Surgery, and the Livingstone University of America (at Charleston, W. Va)., have been annulled, and bills have been introduced into the State Legislature to repeal the charters of the Quaker City Business College, the Penn Medical University, the Philadelphia Electropathic Institution, and the Philadelphia College of Medicine.—*The Record.* (Phila.)

Recently, *Dr. Buchanan* thus spoke of it from his prison cell:

PENN MEDICAL UNIVERSITY.

"Four lecturers in that concern," says Buchanan, "were connected in some way with our Pine street college. One of its professors sold one of our diplomas to a Cuban for $200. Another of its professors Alexander E. E. Falken, is on our list of graduates, although I believe he never took his diploma. W. H. Blake, one of the faculty, is also one of our men."—*The Record* (Phila.)

---

* We read in another paper: "Four bills were presented by Mr. Souder, to annul the charter of the Philadelphia College of Medicine, the charter of the Quaker City Business College, otherwise called the Quaker City College of Applied Sciences, the charter of the Penn Medical College of Philadelphia, otherwise called the Penn Medical University, and the charter of the Philadelphia Electropathic College. These are the institutions of Buchanan fame in connection with the issuing of bogus deplomas."—*The Times,* Philadelphia March 11th, 1881.

Thus we see how disreputable were these institutions and how they continually shifted and changed as though seeking to hide something. But, it will be asked, was the "Penn Medical University" ever, in any sense, a homœopathic medical school? Did it ever, in spite of its character, pretend to teach homœopathy? We answer most emphatically, No! it never even pretended to teach homœopathy. Had it made any pretence at so doing, it could not have done, as only two of its faculty—at the time they were connected with the "University"—pretended to be homœopaths, even in name; and these two lectured, respectively on Botany and Pathological Anatomy. These branches scarcely permit much instruction in the principles and practice of homœopathy, even were the lecturers inclined to such teaching. That they were not so inclined is well proven by their teaching *afterward* in an *avowedly* "electic" college. The historian boastfully says of this "University:" "during the ten years of its existence, its graduates numbered about one hundred and twenty-five, of whom eighty were men and forty-five women. *A large proportion* of these are now practicing homœopathy in different parts of the country."* A large portion!! truly a noble record for a homœopathic medical school. The graduates must have been *well* taught!

Furthermore a list of an *ideal* faculty is given, in this history. We say ideal, for such a faculty never existed as a whole, at one time. It is a piece of patchwork.

We have endeavorod to give a fair statement of the status and purposes of this college and its branches. If any one thinks we have exaggerated in any respect, let him investigate for himself. Let the American Institute of Homœopathy investigate this matter and see if the Penn Medical University deserves to be placed in history as a homœopathic institution. If not, it certainly should not be sent forth under the seal of that body as a homœopathic college. The Institute owes to itself and to the historian, whom we have accused of *perverting the*

* See, History, etc., p. 802. Italics ours.

*history of homœopathy*, that this matter be thoroughly investigated. On the other hand, if the historian considers himself wrongfully accused let him demand an investigation? If we are shown to be in error we will most willingly acknowledge it.

